# Extrinsic and intrinsic drivers of parasite prevalence and parasite species richness in a marine bivalve

**DOI:** 10.1371/journal.pone.0274474

**Published:** 2022-09-26

**Authors:** Kate E. Mahony, Sharon A. Lynch, Xavier de Montaudouin, Sarah C. Culloty

**Affiliations:** 1 School of Biological, Earth and Environmental Sciences, University College Cork, Cork, Republic of Ireland; 2 Aquaculture and Fisheries Development Centre (AFDC), Environmental Research Institute, University College Cork, Cork, Republic of Ireland; 3 MaREI Centre for Climate, Energy and Marine, Environmental Research Institute (ERI), University College Cork, Cork, Republic of Ireland; 4 Université de Bordeaux, CNRS, UMR 5805 EPOC, Station Marine d’Arcachon, Arcachon, France; Swedish University of Agricultural Science, SWEDEN

## Abstract

Parasite species richness is influenced by a range of drivers including host related factors (e.g. host size) and environmental factors (e.g. seawater temperature). However, identification of modulators of parasite species richness remains one of the great unanswered questions in ecology. The common cockle *Cerastoderma edule* is renowned for its diversity and abundance of parasites, yet drivers of parasite species richness in cockles have not been examined to investigate the association of both macro and microparasite communities. Using cockles as a model species, some of the key drivers of parasite prevalence and parasite species richness were investigated. Objectives of this 19-month survey were to determine the influence of the environment, host-parasite dynamics and parasite associations on parasite species richness and prevalence at two different geographic latitudes, chosen based on environmental differences. The highest parasite species richness was recorded in the northern sites, and this was potentially influenced by a range of interactions between the host, the pathogens and the environment. Parasite prevalence increased with host size and age, and parasite species richness increased with reduced salinity. A number of interactions between parasites, and between parasites and pathologies may be influencing parasite infection dynamics. New and concerning information is also presented regarding interactions between parasites and their environment. A number of parasites and potential pathogens (bacteria, *Trichodina* ciliates, metacercariae, trematode sporocysts) may be advantaged under climate change conditions (warming seas, increased precipitation), increasing disease incidence, which may prove detrimental not just for cockles, but for other bivalve species in the future.

## 1. Introduction

Parasites are ubiquitous in the marine environment [1]. Some marine parasite species are topical due to their detrimental effects on the global economy, impacting both wild and reared species [2]. As well as having economic impacts, parasites play important ecological roles. Disease outbreaks are becoming increasingly common in the marine environment [3, 4], and parasitism and disease are likely to be influenced by climate induced variations in temperature, salinity and oxygen [5]. Parasite species richness and biodiversity within a given host is one of the key questions in modern ecology [6]. Despite this, the drivers of parasite species richness is a topic that remains largely unanswered [7]. Across a diverse range of taxa, parasite species richness may be driven by a range of host characteristics including host size, geographical range and population density [6]. For parasite clades with complex life-cycles (e.g. cestodes, trematodes), parasite species richness is also often correlated with free-living diversity in their habitat [8, 9]. However, environmental factors, such as exceeded host tolerance, and those resulting from climate change (e.g. warming seawater, weather extremes) may also influence patterns of parasitism, potentially causing extinctions or conversely increasing species richness in certain areas [10]. Moreover, these climate related influences on parasite-host interactions may create problems due to a cascade through food webs [11–13].

The common cockle *Cerastoderma edule* is a commercially exploited marine bivalve, located along the Eastern Atlantic, from west Russia to west Africa [14–16]. Cockles are ecosystem engineers [17] that form and maintain habitats [18] for species such as *Hydrobia* ([19], as well as influencing hydrodynamics [20, 21]. This species is renowned for its diversity and abundance of parasites, including viruses, bacteria, Microsporidia, Apicomplexa, Ciliophora, Haplosporidia, Turbellaria, Digenea and Crustacea [22–24]. While parasitology in cockles is a centuries old field [25], previously unreported cockle parasites are still being described [26]. Furthermore, novel species, such as the mortality inducing *Marteilia cochillia*, have been reported within the last decade [27]. The impact of parasites infecting cockles may be detrimental depending on parasite species [23], or interactions with poor environmental conditions (e.g. temperature extremes; [23]). Mass mortalities are increasingly reported in cockles, and pathogenic organisms are one of the most commonly reported causes [23, 27–29]. These parasites may also have sub-lethal effects, for example Apicomplexan gregarines, infecting as oocysts, have been linked to destruction of cells within gills, which may contribute to mortality [30].

Large diversity also exists within parasite groups, evidenced by the sixteen documented trematode species, which infect cockles as first (trematode sporocysts; asexual reproductive stage) and secondary (metacercariae; encysted larval stage) intermediate hosts [24, 31]. These can co-occur within a single individual [32, 33]. While not all trematode species found in cockles cause documented negative effects [23], lethal and sublethal impacts have been caused by a number of species [28]. These negative impacts may be worsened due to interaction with unsuitable environmental conditions [34, 35]. As variation in environmental factors occurs between different sites, particularly over large geographical ranges, it is necessary to account for this effect when studying parasite dynamics.

Previous studies of cockles focused on select parasite groups, trematodes in particular, leaving many questions unanswered regarding the interactions between cockle pathogens and driving factors. Few studies investigated coinfection within a clade [36–38]. As an example, coinfection between *Monorchis parvus* and *Gymnophallus choledochus* was lower than expected from mono-infection prevalence [38]. Except for the case hyperparasitism [39–41], studies dealing with coinfection among different parasite clades are even rarer. For example, coinfection between digeneans and *Perkinsus* sp. have been examined, but no association was discovered [42]. Additionally, some previous studies have detailed interactions with the environment in other parasite groups. Interaction between *Vibrio tapetis* (bacteria) and *Himasthla elongata* (trematode) was studied in an experimental cadmium contaminated context [43]. In terms of non-trematode species, *Mycoplasma*-like bacteria can also have detrimental impacts on cockles at higher temperatures [44]. Furthermore, mortality associated Haplosporidians [23] exhibit lower prevalence in areas of reduced salinity [45].

While it appears that parasite studies in cockles often focus on trematodes, this well-studied digenean community makes cockles a suitable model for examining dynamics of parasite-host systems in the climate change scenario [46]. Moreover, cockles are also an appropriate model species for examining both macro and microparasites for a variety of additional reasons. Cockles are often found in intertidal areas [47] and as a result, are likely to come in contact with a wider variety of organisms that can transmit different parasites, such as birds, rather than solely aquatic hosts [23]. Therefore, the dynamics of parasitism are easily studied in cockles due to the potential for contact with a wide array of pathogens. Finally, and importantly for a model species, the cockle is a well-studied, economically and ecologically vital organism, for which it has been deemed a suitable model in previous studies [16, 17, 19].

The overarching aim of this study was to provide knowledge on parasite species richness using a marine bivalve model (the common cockle *Cerastoderma edule*). This study is novel due to the vast geographic range (Ireland to France), the extensive sampling effort (19 months, bimonthly) and the differences in anthropogenic activities (fishing impact, aquaculture, shipping, conservation) at the sample sites. The objectives were to: i) determine parasite species richness across sites, ii) determine if associations exist between the observed parasite groups, and iii) determine if intrinsic (host size) or extrinsic (environmental) factors drive prevalence of pathogens. A number of hypotheses were devised based on previous studies on single parasites/parasite groups. It was expected that species richness would vary depending on host size [36]. Furthermore it was hypothesised that associations would occur between parasites due to the stress of detrimental pathologies, which may increase susceptibility [48, 49]. Similarly it was hypothesised that suboptimal temperature and salinity would result in greater parasite prevalence, due to the impact of stressors on host immune function [50], or impacts to the parasite [45, 51]. This study will provide knowledge on the impact of environmental drivers on parasite prevalence and parasite species richness, and the potential impacts of climate change in the future.

## 2. Materials and methods

### 2.1. Study sites

**Fig 1:**
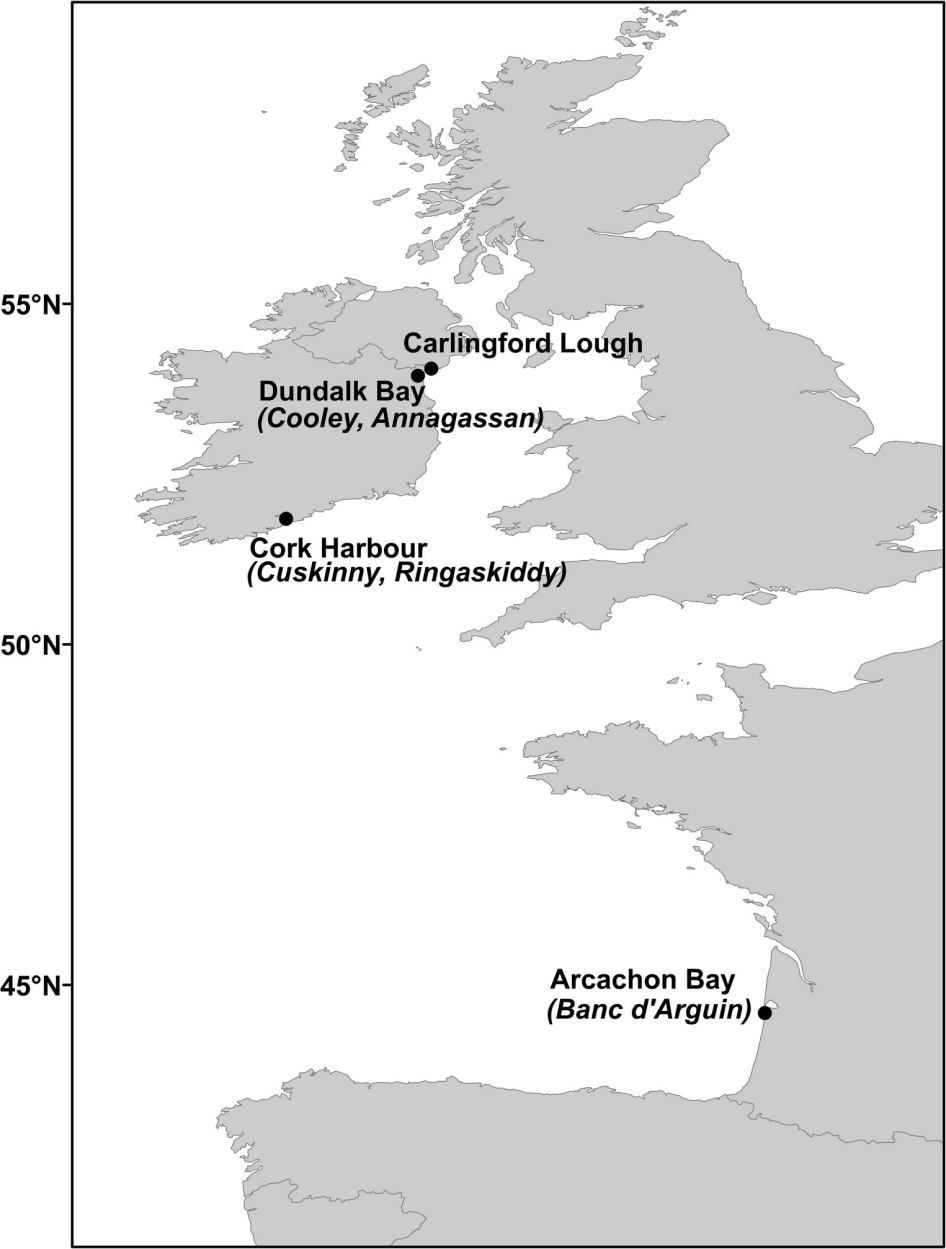
Map of the study sites. In some cases two beds were surveyed within a single site, indicated within brackets. Made with Natural Earth. Free vector and raster map data @ naturalearthdata.com.

The northernmost site was Carlingford Lough (Table 1, Fig 1), which covers an area of 49 km^2^. Samples here were obtained from a cockle bed in the vicinity of a Pacific oyster *Crassostrea gigas* farm. The second Irish study site was nearby at Dundalk Bay (Fig 1, Table 1). The final Irish site examined was Cork Harbour (Fig 1, Table 1), a sheltered bay on the south coast of Ireland. The southernmost sample site was Arcachon Bay in France (Fig 1, Table 1). Cockles *Cerastoderma edule* came from a bed at the 25 km^2^ Banc d’Arguin, a moderately sheltered sandflat located in the south of Arcachon Bay [52].

**Table 1:** Description of the four sites and the beds examined within. Typical activities occurring in the general area of the sites are described.

Site	Bed	Coordinates	Water Quality	Cockle Fishery
Carlingford	Oyster Farm	54°02’N, 6°10’W	Unassigned*	Occasional small scale hand harvesting
Dundalk	Cooley	54°00’N, 6°17’W	Moderate*	Hydraulic suction dredging
	Annagassan	53°52’N, 6°20’W	Good*	
Cork	Cuskinny	51°51’N, 8°15’W	Moderate*	Wild (no commercial fishery)
	Ringaskiddy	51°49’N, 8°18’W	Moderate*	
Arcachon	Banc d’Arguin	44°35’N, 1°14’W	Good*	None†

*Assigned under the Water Framework Directive [53]

†No fishing during the studied period

### 2.2. Sampling

Between April 2018 and October 2019, approximately 30 cockles were collected from each bed, every other month, i.e. for sites with two distinct beds c. 60 cockles were collected on each occasion. Sites were chosen to account for a variation in the latitudinal range of cockles. Sampling was carried out opportunistically, with both hand collection and raking of buried and surfaced cockles, depending on sampling constraints. At Arcachon, sampling ceased in June 2019, due to mass mortality of cockles in the area. Lower numbers of approximately 20 per sample were obtained in Cork due to low densities and rocky substrate.

### 2.3. Histology

In total, 1,353 cockles were examined histologically (Carlingford = 229, Dundalk = 478, Cork = 407, Arcachon = 239). Prior to dissection for histology, the whole weight (including shell, dried with laboratory paper) and length of each individual was obtained. Cockles lay down rings each winter [54]. These rings were counted as an estimation of age, with each ring equivalent to a year’s growth.

For histology, large individuals were divided and the tissue fixed included a representation of the major tissue groups (mantle, visceral mass, digestive system, foot, gill). Smaller cockles were placed whole into tissue embedding histocassettes. After 24–48 hours in Bouin’s solution (Arcachon samples) or Davidson’s solution (all other samples) [55], the tissues were prepared for paraffin embedding by undergoing a 20-hour cycle through graded ethanol volumes, adapted from [56]. Finally, slides were prepared by sectioning the embedded tissue to at least 5 μm, followed by staining with Haematoxylin and Eosin [57]. Screening for pathologies, macroparasites, and microparasites [23] was conducted using a NikonEclipse 80i light microscope, at 4X, 10X and 40X. Presence or absence of internal parasites and lesions were recorded for each individual.

### 2.4. Statistical analysis

#### 2.4.1. Patterns of parasite species richness

In this study, total species richness for each site was determined as the total number of parasite species recorded, over the sampling period. Mean species richness per individual was calculated as an average species number per individual cockle. Species richness in this study actually refers to minimum species richness, as in some cases it was impossible to classify parasites to species level. While a variety of indices can be used to describe species patterns, this method of species richness was chosen for easy comparison with previous studies of parasites in cockles (e.g. [52, 58]). Kruskal Wallis tests (following assessment of normality and homogeneity of variance) were employed to determine if the species richness differed across beds. Post hoc Dunn tests were applied if a variable was significant, to determine which beds differed from each other. The relationship between individual species richness with length was assessed using linear regression.

#### 2.4.2. Associations between parasites

Probabilistic species co-occurrence analysis was conducted in R using the ‘cooccur’ package [59] to determine if associations existed between parasites, and between parasites and pathologies. The ‘cooccur()’ function within this package examines all pairwise combinations to determine the probability of these combinations co-occurring more or less frequently than expected.

#### 2.4.3. Site related differences of environmental variables

Environmental data (dissolved oxygen, sea temperature and salinity) were obtained from the Atlantic-Iberian Bay Irish-Ocean Physics Analysis and Forecast [60]. Previous studies have successfully used oceanogeographic modelling to assess the influence of environmental variables [45, 61, 62] and these data also agreed with other published data from the study sites [63]. All analyses were conducted using R Version 1.2.5033 [64]. The difference between environmental variables across beds was determined using Kruskal Wallis and Dunn tests.

#### 2.4.4. Relationship between intrinsic and extrinsic variables, and parasite prevalence

To determine if environmental variables (sea temperature, dissolved oxygen, salinity), site or host length influenced parasite prevalence, binomial generalised linear models were applied, using the ‘lme4’ package in R [65]. Models were run separately for each key parasite/parasite group. Prior to testing, explanatory variables with correlations greater than ±0.5 were omitted due to high collinearity. Additionally, due to the possibility of autocorrelation between length and age, as well as the absence of age data for 106 individuals, age was not included as an explanatory variable in these models. Sites were included as contrasts to examine differences between locations.

## 3. Results

### 3.1. Patterns of parasite species richness

A variety of both macro and microparasites (S1 Table) were observed within cockles *Cerastoderma edule* in this study. Macroparasites included Crustacea, fungi (in one cockle), trematodes (*Gymnophallus minutus* and unidentified metacercariae and trematode sporocysts) and Turbellaria (*Paravortex* spp.). Microparasites included Apicomplexa (Coccidia and Gregarina), bacteria (in the gill and digestive gland), ciliates (*Trichodina-*like and *Rhynchodida*-like) and Haplosporidia. Haplosporidian infection (observed as sporonts in the connective tissue) was detected at all sites, except Arcachon. Pathologies were also observed during sampling. Granulomas were observed at all sites, typically in the gills or mantle. A low prevalence of necrosis was detected at Carlingford, Dundalk and Cork. Finally, neoplasia was observed at all sites with the exception of Dundalk.

**Fig 2:**
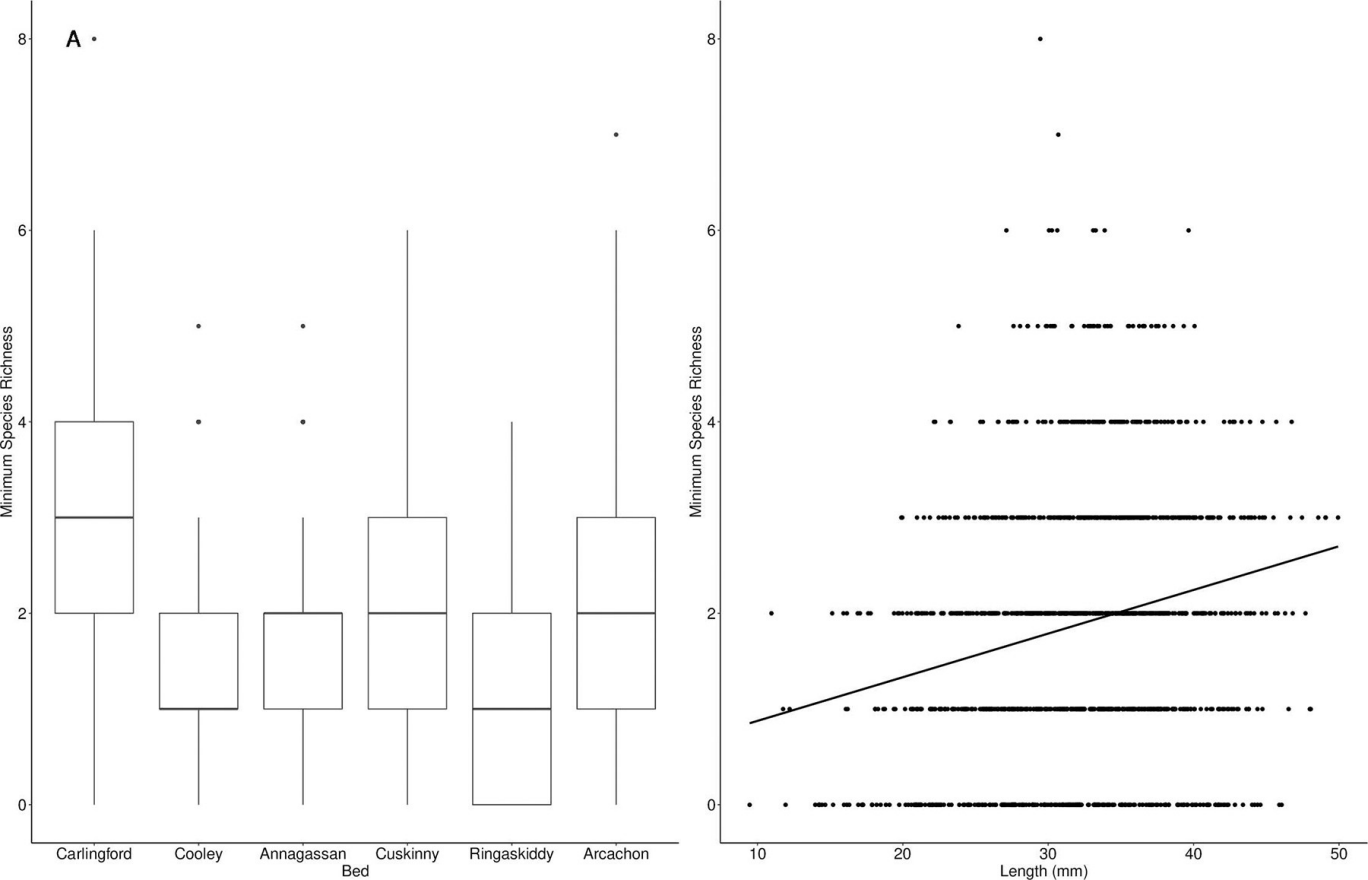
Relationships between minimum parasite species richness (not all identified to species level) and (A) cockle beds and (B) cockle length, between April 2018 and October 2019. Boxplots in (A) demonstrate the median values, boxes extend from the 25^th^ to 75^th^ percentile of each group’s distribution of values, and whiskers show the largest and smallest values within 1.5 times the interquartile range. Outliers are indicated by individually plotted points.

Total parasite species richness was similar at each of the sites (Carlingford = 11, Dundalk = 13, Cork = 12, Arcachon = 10). Median individual parasite species richness differed significantly across beds (*H* = 273.14, *df* = 5, *p* < 0.001, Fig 2A). A post hoc Dunn test found that individual species richness was highest at Carlingford (3.24 ± 1.28), compared with all other sites (*p* < 0.001 in all cases). When examining beds within the same site, parasite species richness differed significantly between Ringaskiddy, Cork (1.16 ± 1.24; mean ± SD) and Cuskinny, Cork (1.77 ± 1.24, *p* < 0.001). Individual parasite species richness also increased significantly in longer (*F* = 64.67, *p* < 0.001, Fig 2B) and older cockles (*F* = 82.15, *p* < 0.001). However the adjusted R^2^ was 0.05 and 0.06 respectively, indicating that a large proportion of the variance in species number was not explained by length or age.

### 3.2. Associations between pathogens

#### 3.2.1. Association between pathogens and pathologies

**Fig 3:**
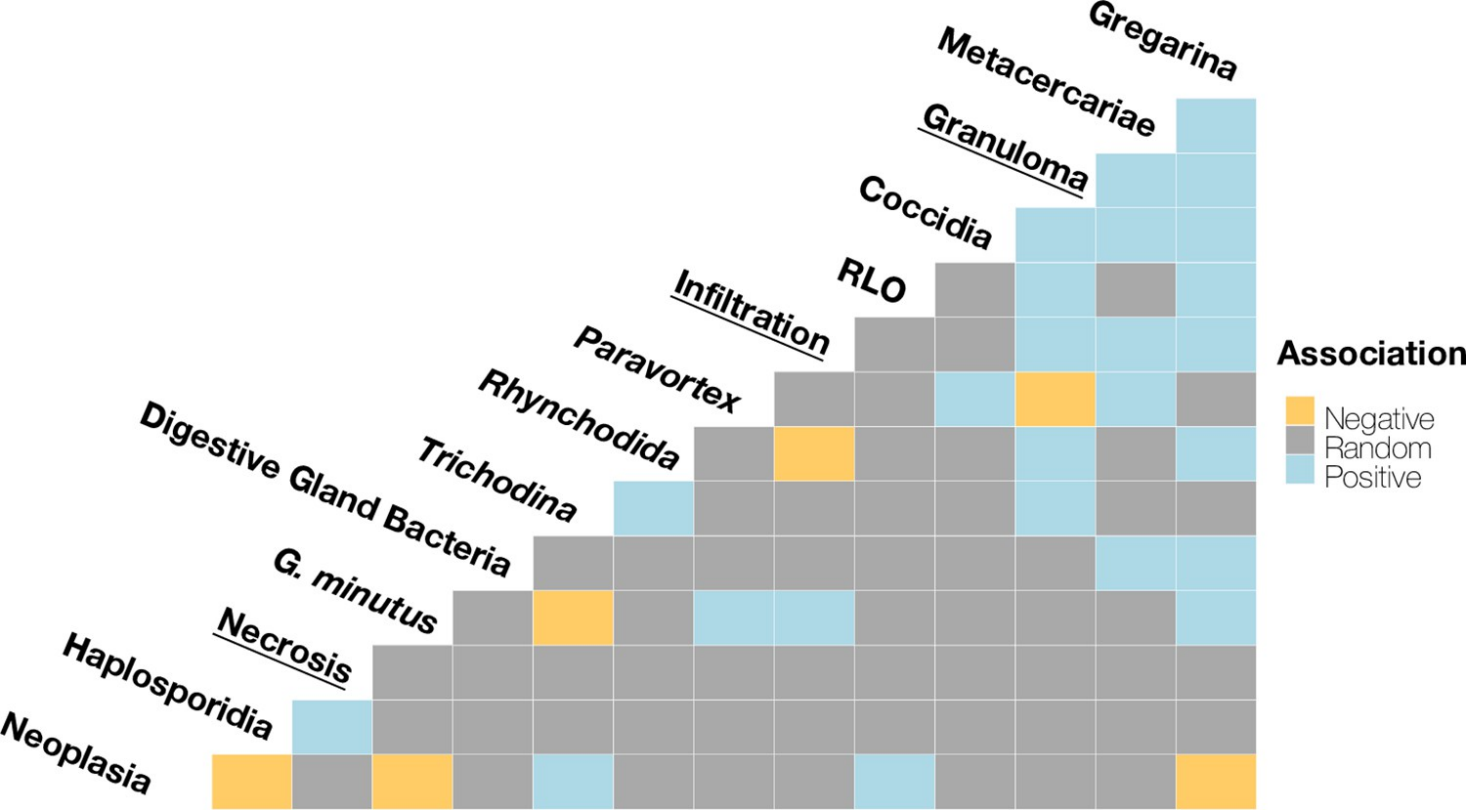
Heat map demonstrating a species co-occurrence matrix, showing relationships between parasite species and cockle pathologies (underlined) as determined by a probabilistic co-occurrence model from the “cooccur” package in R. Species names indicate the pairwise comparisons made.

Following species co-occurrence analysis, some significant associations were detected, of which 25% were positive and 6% were negative. The remainder of the pairs (69%) were random. As a threshold was applied to remove pairs which co-occurred once, 16.7% of pairs were removed from the analysis (Fig 3). A number of associations were detected between pathogens and cockle pathologies (Fig 3). A positive association was detected between granulomas and *Rickettsiae*-like infection (*p* = 0.014), gregarines (*p* < 0.001), *Trichodina* ciliates (*p* = 0.018), *Rhynchodida*-like ciliates (*p* < 0.001), Coccidia (*p* = 0.035) and metacercariae (excluding *G*. *minutus*, *p* < 0.001). A negative association was detected between *Paravortex* and granulomas (*p* = 0.042). Necrosis was only associated with one pathogen, Haplosporidia (*p =* 0.044), where necrosis was more likely in infected individuals. Infiltration of haemocytes was positively associated with granulomas (*p* = 0.025), gregarines (*p* < 0.001) and metacercariae (*p* < 0.001 for unclassified metacercariae and *G*. *minutus*). A negative association was observed between infiltration and *Rhynchodida*-like ciliates (*p* = 0.024). Finally, neoplasia was negatively associated with gregarines (*p* < 0.001), *G*. *minutus* (*p* = 0.001) and Haplosporidians (*p* = 0.028). A positive association was detected between *Rickettsiae*-like infection and neoplasia (*p* = 0.024), as well as neoplasia and *Trichodina* ciliates (*p* = 0.042).

#### 3.2.2. Associations between parasites

A number of parasite species were found to co-occur more frequently than expected. Gregarines were positively associated with detection of bacterial foci in the digestive system (*p* = 0.008), *Rickettsiae*-like infection (*p* = 0.009), *Rhynchodida*-like ciliates (*p* = 0.010), Coccidia (*p* = 0.004) and metacercariae (*p* < 0.001), including *Gymnophallus minutus* (*p* < 0.001). An association existed between bacterial foci in the digestive system and the detection of metacercariae (*p* < 0.001, not *G*. *minutus*). Those infected with *G*. *minutus* were likely to also be infected with *Paravortex* spp. (*p* = 0.016). A positive association also existed between *Trichodina* ciliates and *Rhynchodida*-like ciliates (*p* = 0.015), and between metacercariae (not *G*. *minutus*) and *Paravortex (p* = 0.005). A negative interaction was detected between *Trichodina* and *G*. *minutus* (*p* = 0.004). Positive associations were observed between Coccidia and metacercariae (*p* < 0.001), and between Coccidia and *Paravortex* (*p* = 0.038).

### 3.3. Site related differences in environmental variables

**Fig 4:**
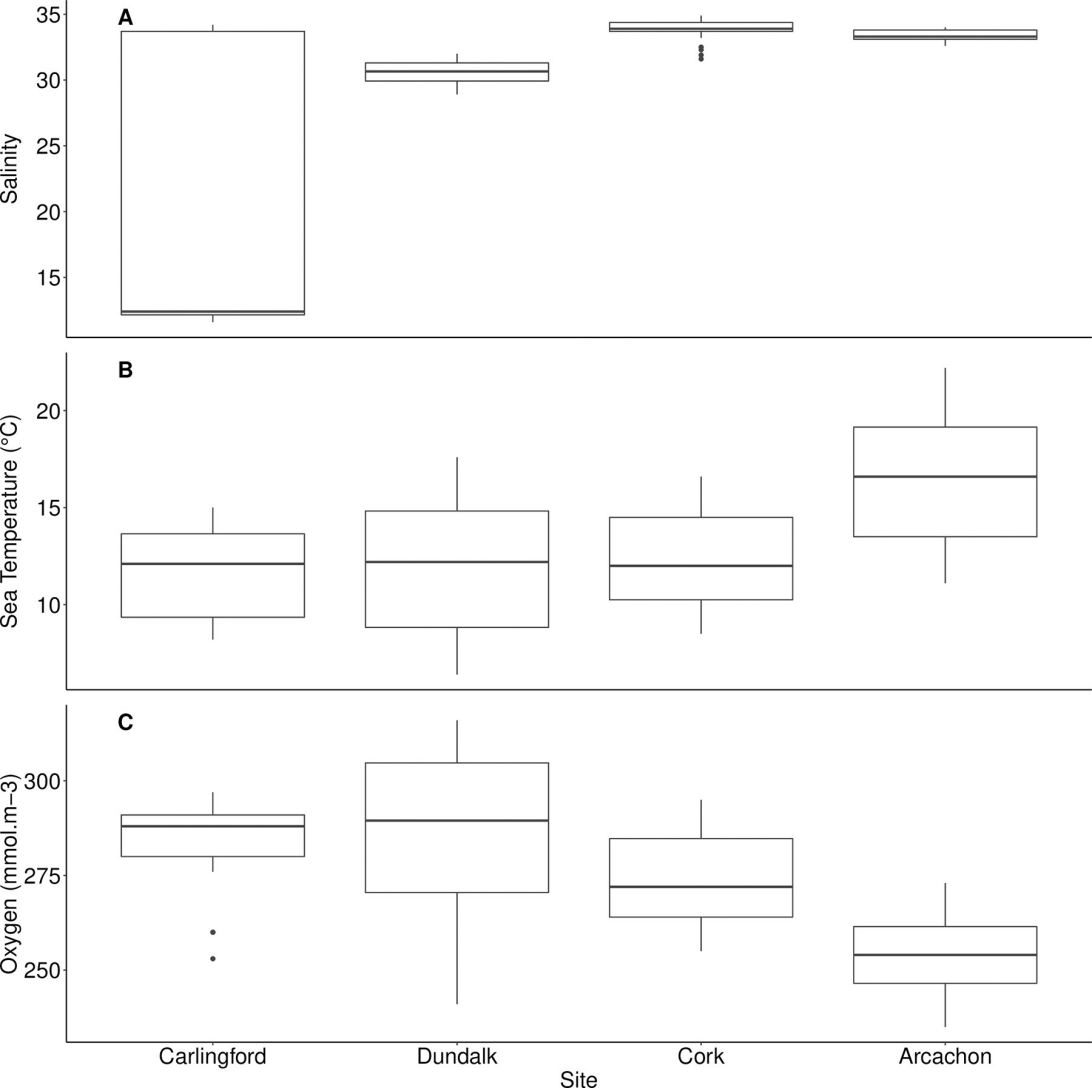
Boxplots describing differences in (A) salinity, (B) sea temperature and (C) dissolved oxygen for each of the sites surveyed for histological analysis of *Cerastoderma edule*. The boxplots display the median values, with boxes extending from the 25^th^ to 75^th^ percentile of each group’s distribution of values, and whiskers demonstrate the largest and smallest values within 1.5 times the interquartile range. Outliers are indicated by individually plotted points.

Salinity differed significantly between beds (*H* = 66.61, *df* = 5, *p* < 0.001, Fig 4A). Salinity at Carlingford was significantly lower than all other beds (19.8 ± 10.4; mean ± SD), with the exception of the beds at Dundalk (*p* < 0.001 in all cases, Annagassan = 30.6 ± 0.8, Cooley = 30.6 ± 0.7). However, the salinity range was much greater at Carlingford, compared with these sites.

Sea temperature differed significantly between beds (*H* = 18.75, *df* = 5, *p* = 0.002, Fig 4B). While temperatures did not differ between the Irish beds, seawater at Arcachon (16.3°C ± 3.5, mean ± SD) was significantly warmer than all the Irish sites (*p* < 0.001, with the exception of Cuskinny: 12.4°C ± 2.48 SD), where a trend existed (*p* = 0.028 with a significance level of 2.5% to correct for pairwise analysis).

Finally, oxygen levels differed significantly between beds (*H* = 37.70, *df* = 5, *p* < 0.001, Fig 4C). As was the case with temperature, Irish beds did not differ significantly. However, dissolved oxygen at Arcachon (254 mmol/m^3^ ± 10.8; mean ± SD) was significantly lower than all of the Irish sites (*p* < 0.025 in all cases).

### 3.4. Relationship between intrinsic and extrinsic variables, and parasite prevalence

#### 3.4.1. Cockle length and parasite prevalence

**Fig 5:**
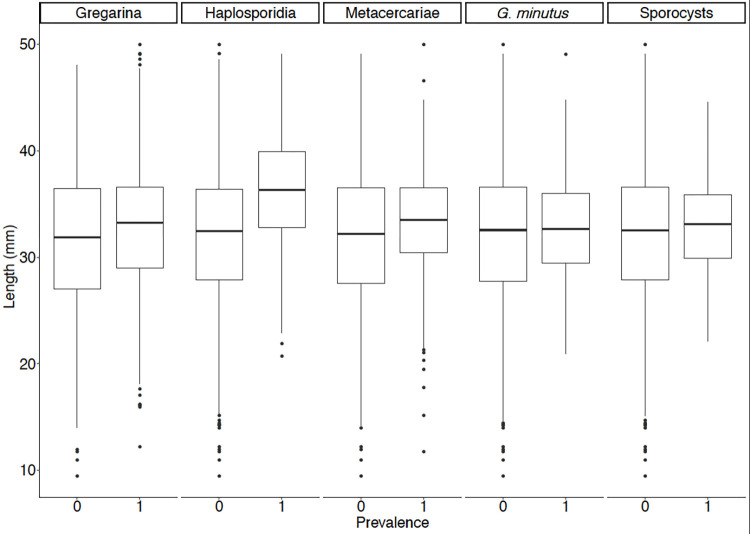
Boxplots demonstrating the relationship between pathogens and cockle shell length. Absence of pathogens is indicated by 0 and presence is indicated by 1. The boxplots display the median values, boxes extend from the 25^th^ to 75^th^ percentile of each group’s distribution of values, and whiskers show the largest and smallest values within 1.5 times the interquartile range. Outliers are indicated by individually plotted points.

Gregarines (likely *Nematopsis* sp.) were the most commonly observed parasite, being observed mainly in the gills and mantle, but also across all tissue groups. Results from binomial generalised models indicate that gregarine infection was more likely to occur in larger host individuals (*z* = 2.96, *p* = 0.004, Fig 5). A number of other parasites were more likely to infect larger individuals: Haplosporidians (*z* = 3.81, *p* < 0.001, Fig 5), *G*. *minutus* (*z* = 2.27, *p* = 0.023), metacercariae (*z* = 3.07, *p* = 0.002) and trematode sporocysts (*z =* 2.44, *p* = 0.015, Fig 5).

#### 3.4.2. Environmental variables and parasite prevalence

**Fig 6:**
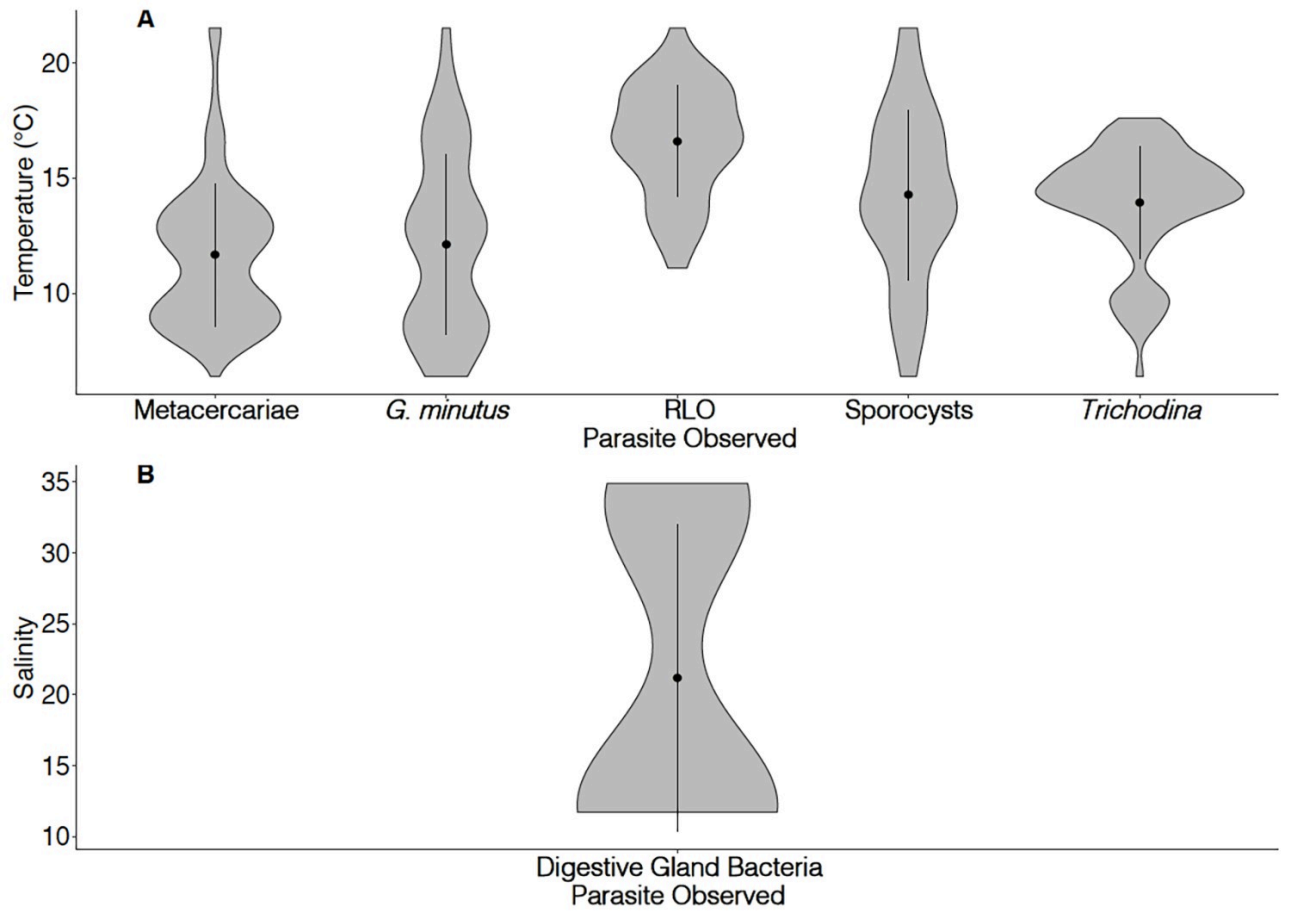
Violin plots (visualising the kernel probability density), demonstrating the relationship between the presence of pathogens and (A) seawater temperature and (B) salinity, at all sites combined. Mean ± 1 SD of salinity/temperature is represented by the vertical line and point within the violin plots.

Binomial generalised linear model outputs (previously discussed in section 3.4.1.), show that a range of parasites are more likely to occur at higher temperatures (Fig 6A): *Rickettsiae*-like infection (*z* = 6.36, *p* < 0.001), *Trichodina* ciliates (*z* = 5.23, *p* < 0.001), and trematode sporocysts (*z* = 2.07, *p* = 0.004). Additionally, trematode sporocyst prevalence was highest at the warmest site, Arcachon (*z =* 2.41, *p =* 0.041). *G*. *minutus* and other metacercariae were more likely to occur at lower temperatures (*z* = -3.51, *p* < 0.001 and *z* = -2.11, *p* = 0.004 respectively).

Similarly, an association was detected with salinity. Bacterial foci in the digestive gland epithelium were more likely at lower salinities (*z* = -4.52, *p* < 0.001, Fig 6B). Carlingford, the site with the lowest salinity demonstrated highest prevalence of Coccidian infection of the kidney and the intestine, gregarines and metacercariae (results of contrasts detailed in S6 Table). However, site wise differences were not always related to environmental variations. For example in *G*. *minutus*, prevalence was lower in Cork and higher in Arcachon, compared with Carlingford (S6 Table).

## 4. Discussion

Using the common cockle *Cerastoderma edule* as a model host species, this study provides new insights into the modulators of parasite prevalence and parasite species richness in bivalves. It was found that size of the host, as well as environmental factors (in particular salinity and temperature) both drive or inhibit parasite species and prevalence. Furthermore it was shown that parasite species richness may also be influenced by interactions between pathogens. The high species richness demonstrated in cockles, the model species, affirms its important role as host to a diverse array of parasites. Overall parasite species richness at Dundalk, one of northern sites was potentially greater than 13, due to the method of trematode identification, exceeding that of previous findings [66]. This represents one third of the total taxons known to infect cockles [24]. This high species richness is influenced by a combination of extrinsic (environmental) and intrinsic (host size) factors.

This study confirmed that the intrinsic drivers of host size (and potentially age due to the correlation) played a role in parasite prevalence and parasite species richness. Previously in cockles, the relationship between parasite prevalence and host size has been demonstrated in few parasite groups. For example, cockle length and trematode sporocyst prevalence demonstrated a positive correlation [37], which was supported by this study. A positive size effect on *G*. *minutus* was observed, which contradicts [41]. This size dependent effect was not isolated to trematodes, with gregarines and Haplosporidia also more likely to occur in larger individuals (the latter in agreement with [45]). The findings of this study support previous findings of a positive size effect on trematode richness in cockles, with adults displaying greater species richness [58]. For trematodes, increased water clearance and longer exposure time in larger (and likely older cockles) increase the likelihood of infections [67]. Thus, it is possible that similar effects are increasing the species richness of both macro and microparasites.

Environmental characteristics were found to be an important driver of parasite prevalence. Higher seawater temperatures correlated with increased prevalence of *Rickettsiae*-like infection, *Trichodina* ciliates, metacercariae and trematode sporocysts, while low salinity corresponded with increased prevalence of bacterial foci. In terms of the host, higher temperatures are associated with increased filtration [68], which may further result in increased exposure to parasites. In terms of the parasite, this could be related to seasonal variations in parasite dynamics. While it is important to note that more sites should be examined to determine these effects, these potential relationships are concerning, considering the likelihood of increased water temperatures and reduced salinity (due to increased precipitation), resulting from climate change in many regions [69]. It is therefore possible that these parasites may be advantaged in a changing climate, causing problems due to the mortality inducing potential of some parasite species [23].

Associations were observed between a number of parasite species. These associations may be a direct result of the relationship between pathogens, or may be attributed to the environmental characteristics of the sites, promoting co-infection. These associations may influence the impact of any stressor, including climate change, on cockles. Due to the possibility of some parasites facilitating infection by others, climate change may have far reaching consequences. For example, metacercariae and bacterial foci infections are likely to be impacted by increased water temperature or reduced salinity. However these parasites are often associated with infection by gregarines, which have been linked to mortalities [30]. Therefore, while gregarines were not found to be influenced by environmental factors, climate change may indirectly influence the prevalence of this parasite group.

Further interactions between pathogens and the host were evident in the pathologies observed. A particularly problematic pathology noted was disseminated neoplasia, which is linked to immunosuppression and provoke mortality in cockles [48, 49]. In the individuals studied, *Rickettsiae*-like infection of the gills was more likely in neoplasia impacted cockles. However negative associations were also discovered relating to neoplasia, most notably between neoplasia and Haplosporidian infection (i.e. Haplosporidian infection was less likely in cockles with neoplasia), despite combinations of this pathogen/pathology being previously attributed to mass mortality [66]. The fact that neoplasia was absent from Dundalk, despite relatively high Haplosporidian infection, indicates that Haplosporidian infection is not linked to the aetiology of neoplasia. Furthermore in terms of neoplasia, prevalence at Cork and Arcachon was reduced since the 1980s and 2000s respectively [70, 71]. However, as these are just two time points, it is impossible to determine if prevalence fluctuated in the interim. These associations, along with the remaining associations discovered in this study, should be analysed experimentally to confirm these interactions, or determine if they are associated with changing climate and disease cycles.

Stressors in the environment lead to stress in shellfish, which in turn results in immunosuppression and increased disease risk [50]. In agreement with previous studies [72–74], low salinity was confirmed as one of the most stressful factors influencing cockles. This was most evident at Carlingford, which exhibited high overall parasite species richness and greatest individual species richness. Two conflicting scenarios may explain the impact of salinity on parasite dynamics in cockles. First, salinity may cause valve closure to prevent osmotic shock, resulting in reduced respiration [74]. Second, and conversely, stressful conditions may cause cockles to increase respiration, following depletion of initial energy reserves [75]. The second scenario (delayed increase in respiration) is most likely, considering cockles at Carlingford exhibit reduced growth in later years [76], possibly due to energy allocation to respiration. Therefore, it appears that cockles at Carlingford, a stressful environment, allocate energy to homeostasis, rather than immune function, potentially contributing to greater parasite species richness. This increased respiration may also facilitate transmission in a mechanical manner, with parasites gaining access through increased water entering the organism via the inhalant current. Additionally, cockles at Carlingford are larger than at the nearby site of Dundalk, [76], and these larger sizes may be driving species richness at Carlingford. For example, larger cockles have had higher metacercarial infection levels in previous investigations [77]. Diversity of host species is also an important driver of trematode parasite diversity [8], and Carlingford Lough is a site of oyster and mussel culture, which usually stimulates biodiversity [78]. Previously Turbellaria and trematodes have been identified at this site [79]. However diversity of potential hosts (birds, fish, invertebrates) was not quantified, therefore it was not possible to verify if other species are the key influencer. Similarly, fishing had no detectable impact on parasite species richness, as no patterns were evident between harvested and wild areas.

This study provides an insight into the drivers of parasite species richness in bivalves, which are a complex combination of factors. Previously known relationships between size in certain parasite groups were affirmed, but evidence of this relationship was also demonstrated in gregarines. While the confounding factor of similar environments may lead to the co-presence of certain pathogens at a given site, it was probable that interactions between pathogens are a driver of infection, providing an important starting point for future experimentation. Low salinity was affirmed as a stressor, causing increased parasite species richness and prevalence. However, most importantly in terms of the environment, this study provides new insights into the potential impacts of climate change on bivalve parasite dynamics. It appears that not all parasite groups will be similarly impacted by climate change. Due to high temperatures and increased precipitation resulting in reduced salinity [69], it is likely that some parasites may be advantaged (bacterial inclusions, *Trichodina* ciliates, metacercariae, trematode sporocysts, gregarines), either by direct or indirect effects. This is likely to have cascading impacts on cockles, their predators and the wider ecosystem. Therefore, future interactions between bivalve hosts, their parasites and the environment must be closely monitored in order to minimise potentially detrimental impacts on hosts and their ecosystems.

## Supporting information

S1 Table Prevalence of key observed species and pathological conditions (lesions). Data obtained at three sites in Ireland, and one site in France (Arcachon), between April 2018 and October 2019. Additionally, a *Sphenophyra*-like ciliate was detected in one individual in Dundalk and fungus was detected in another individual in Dundalk.
(DOCX)

S2 Table Results of a Dunn test comparing individual parasite species richness by bed. (DOCX)

S3 Table Results of a Dunn test comparing salinity by bed. (DOCX)

S4 Table Results of a Dunn test comparing seawater temperature by bed. (DOCX)

S5 Table Results of a Dunn test comparing oxygen by bed. (DOCX)

S6 Table Results of significant contrasts examined within a binary generalised linear model, examining the impact of intrinsic and extrinsic variables on prevalence of parasites in *C*. *edule*. (DOCX)

S1 Fig Examples of pathogens and pathologies (arrows) observed in *Cerastoderma edule* from Ireland and France, between April 2018 and October 2019. Slides were prepared using histological techniques and stained with Haematoxylin and Eosin. (A) *Rickettsiae*-like infection in the gill (B) Gregarines within a granuloma (C) *Trichodina* ciliate external to the gill (D) Trematode metacercariae in the foot (E) *Gymnophallus minutus* in the hinge tissue (F) Disseminated neoplasia in the connective tissue.
(DOCX)

S2 Fig Violin plots (visualising the kernel probability density), demonstrating the relationship between the presence of pathogens and seawater temperature, at each of the sites. Mean ± 1 SD of temperature is represented by the vertical line and point within the violin plots.
(DOCX)

S3 Fig Violin plots (visualising the kernel probability density), demonstrating the relationship between the presence of pathogens and salinity, at each of the sites. Mean ± 1 SD of salinity is represented by the vertical line and point within the violin plots.
(DOCX)
