# Supplementary material for: Extrinsic and intrinsic drivers of parasite prevalence and parasite species richness in a marine bivalve
Source: PLoS One. 2022 Sep 26;17(9):e0274474. doi: 10.1371/journal.pone.0274474 (PMC9512183; doi:10.1371/journal.pone.0274474)
Supplement: S1 Table — Data obtained at three sites in Ireland, and one site in France (Arcachon), between April 2018 and October 2019. Additionally, a Sphenophyra-like ciliate was detected in one individual in Dundalk and fungus was detected in another individual in Dundalk. (DOCX) [file pone.0274474.s001.docx]

**Supplementary Material: Extrinsic and intrinsic drivers of parasite prevalence and parasite species richness in a marine bivalve**

**S1 Table. Prevalence of key observed species and pathological conditions (lesions)**. Data obtained at three sites in Ireland, and one site in France (Arcachon), between April 2018 and October 2019. Additionally, a *Sphenophyra*-like ciliate was detected in one individual in Dundalk and fungus was detected in another individual in Dundalk.

| Prokaryotes |  | | **Carlingford** | | **Dundalk** | | **Cork** | | **Arcachon** | |
| --- | --- | --- | --- | --- | --- | --- | --- | --- | --- | --- |
|  |  |  | Prevalence (%) | CI | Prevalence (%) | CI | Prevalence (%) | CI | Prevalence (%) | CI |
|  | Gill |  | 0.00 | 0.00-0.02 | 0.33 | 0.00-0.02 | 0.74 | 0.00-0.02 | 11.72* | 0.08-0.16 |
|  | Digestive Gland |  | 4.80* | 0.02-0.08 | 0 | 0.00-0.01 | 1.23 | 0-0.03 | 1.26 | 0.00-0.04 |
| Protozoa | Apicomplexa | Coccidia | 7.42* | 0.04-0.12 | 0.84 | 0.00-0.02 | 0.98 | 0-0.02 | 2.09 | 0.01-0.05 |
|  |  | Gregarina | **87.34*** | 0.82-0.91 | **64.02** | 0.60-0.68 | **33.42** | 0.29-0.38 | **45.61** | 0.39-0.52 |
|  | Ciliates | Trichodina | 3.49 | 0.02-0.07 | 4.18 | 0.03-0.06 | 12.29* | 0.09-0.16 | 1.26 | 0.00-0.04 |
|  |  | Rhynchodida | 6.99 | 0.04-0.11 | 4.18 | 0.03-0.06 | 11.79* | 0.09-0.15 | 2.51 | 0.01-0.05 |
|  | Haplosporidia |  | 6.99* | 0.04-0.11 | 3.56 | 0.02-0.06 | 4.18 | 0.02-0.07 | 0.00 | 0.00-0.02 |
| Metazoa | Trematode | Metacercariae | 82.53* | 0.77-0.87 | 1.05 | 0.00-0.02 | 12.53 | 0.09-0.16 | 20.08 | 0.15-0.26 |
|  |  | Sporocyst | 4.80 | 0.02-0.08 | 4.80 | 0.02-0.08 | 2.95 | 0.02-0.05 | 12.13* | 0.08-0.17 |
|  |  | *Parvatrema* | 15.72 | 0.11-0.21 | 17.57 | 0.14-0.21 | 1.97 | 0.01-0.04 | 18.83* | 0.14-0.24 |
|  | Crustacea | Unidentified | 0.44* | 0-0.02 | 0.42 | 0.00-0.02 | 0.25 | 0-0.01 | 0.00 | 0.00-0.02 |
|  | Turbellaria | *Paravortex* | 10.04 | 0.06-0.15 | 2.93 | 0.02-0.05 | 3.19 | 0.02-0.05 | 27.62* | 0.22-0.34 |
|  | Total Species Richness | | 11 | | 14 | | 12 | | 10 | |
|  | Sample Size | | 229 | | 478 | | 407 | | 239 | |
|  | Mean Individual Species Richness (±1 SD) | | 3.24 ± 1.28 | | 1.54 ± 1.1 | | 1.52 ± 1.27 | | 1.93 ± 1.39 | |
| Lesions | Infiltration |  | 23.58* | 0.18-0.30 | 13.6 | 0.11-0.17 | 8.35 | 0.06-0.11 | 13.81 | 0.10-0.19 |
|  | Granuloma |  | **67.25*** | 0.61-0.73 | **32.22** | 0.28-0.37 | **36.36** | 0.32-0.41 | **29.29** | 0.24-0.36 |
|  | Necrosis |  | 2.18 | 0.01-0.05 | 3.94* | 0.02-0.06 | 3.19 | 0.02-0.05 | 0.00 | 0.0-0.02 |
|  | Neoplasia |  | 0.44 | 0.00-0.01 | 0 | 0.00-0.01 | 18.43* | 0.15-0.23 | 6.7 | 0.04-0.11 |

For each sample site the parasite species with the highest % prevalence is denoted in bold font, * denotes the highest % prevalence for each parasite species or pathological condition.
