# Supplementary material for: Extrinsic and intrinsic drivers of parasite prevalence and parasite species richness in a marine bivalve
Source: PLoS One. 2022 Sep 26;17(9):e0274474. doi: 10.1371/journal.pone.0274474 (PMC9512183; doi:10.1371/journal.pone.0274474)
Supplement: S2 Table — (DOCX) [file pone.0274474.s002.docx]

**Supplementary Material: Extrinsic and intrinsic drivers of parasite prevalence and parasite species richness in a marine bivalve**

**S2 Table. Results of a Dunn test comparing individual parasite species richness by bed.**

|  | **Annagassan** | **Arcachon** | **Carlingford** | **Cooley** | **Cuskinny** |
| --- | --- | --- | --- | --- | --- |
| Arcachon | 0.0425 |  |  |  |  |
| Carlingford | **<0.001** | **<0.0001** |  |  |  |
| Cooley | 0.9999 | 0.0514 | **<0.0001** |  |  |
| Cuskinny | 0.4859 | 0.9999 | **<0.0001** | 0.5581 |  |
| Ringaskiddy | **0.0179** | **<0.0001** | **<0.0001** | **0.0148** | **<0.0001** |
