# Supplementary material for: Extrinsic and intrinsic drivers of parasite prevalence and parasite species richness in a marine bivalve
Source: PLoS One. 2022 Sep 26;17(9):e0274474. doi: 10.1371/journal.pone.0274474 (PMC9512183; doi:10.1371/journal.pone.0274474)
Supplement: S3 Table — (DOCX) [file pone.0274474.s003.docx]

**Supplementary Material: Extrinsic and intrinsic drivers of parasite prevalence and parasite species richness in a marine bivalve**

**S3 Table. Results of a Dunn test comparing salinity by bed.**

|  | **Annagassan** | **Arcachon** | **Carlingford** | **Cooley** | **Cuskinny** |
| --- | --- | --- | --- | --- | --- |
| Arcachon | **0.0031** |  |  |  |  |
| Carlingford | 0.9999 | **0.0041** |  |  |  |
| Cooley | 0.9999 | **0.0031** | 0.9999 |  |  |
| Cuskinny | **<0.0001** | 0.6947 | **<0.0001** | **<0.001** |  |
| Ringaskiddy | **<0.0001** | 0.9057 | **<0.0001** | **<0.001** | 0.9999 |
