# Supplementary material for: Extrinsic and intrinsic drivers of parasite prevalence and parasite species richness in a marine bivalve
Source: PLoS One. 2022 Sep 26;17(9):e0274474. doi: 10.1371/journal.pone.0274474 (PMC9512183; doi:10.1371/journal.pone.0274474)
Supplement: S5 Table — (DOCX) [file pone.0274474.s005.docx]

**Supplementary Material: Extrinsic and intrinsic drivers of parasite prevalence and parasite species richness in a marine bivalve**

**S5 Table. Results of a Dunn test comparing oxygen by bed.**

|  | **Annagassan** | **Arcachon** | **Carlingford** | **Cooley** | **Cuskinny** |
| --- | --- | --- | --- | --- | --- |
| Arcachon | **<0.0001** |  |  |  |  |
| Carlingford | 0.9999 | **<0.0001** |  |  |  |
| Cooley | 0.9999 | **<0.0001** | 0.9999 |  |  |
| Cuskinny | 0.9999 | **0.0140** | 0.8629 | 0.1468 |  |
| Ringaskiddy | 0.9999 | **0.0137** | 0.8757 | 0.1497 | 0.9999 |
