# Supplementary material for: Extrinsic and intrinsic drivers of parasite prevalence and parasite species richness in a marine bivalve
Source: PLoS One. 2022 Sep 26;17(9):e0274474. doi: 10.1371/journal.pone.0274474 (PMC9512183; doi:10.1371/journal.pone.0274474)
Supplement: S6 Table — (DOCX) [file pone.0274474.s006.docx]

**Supplementary Material: Extrinsic and intrinsic drivers of parasite prevalence and parasite species richness in a marine bivalve**

**S6 Table**. **Results of significant contrasts examined within a binary generalised linear model, examining the impact of intrinsic and extrinsic variables on prevalence of parasites in *C. edule*.** Four sites were included as factors, with Dundalk Bay, Cork Harbour and Arcachon compared with Carlingford Lough, for each parasite or parasite group.

| **Parasite** | **Site** | ***z*** | ***p*** |
| --- | --- | --- | --- |
| Coccidia | Dundalk | -4.008 | <0.001 |
|  | Cork | -3.717 | <0.001 |
|  | Arcachon | -2.556 | 0.016 |
| Gregarina | Dundalk | -6.010 | <0.001 |
|  | Cork | -11.582 | <0.001 |
|  | Arcachon | -8.139 | <0.001 |
| Metacercariae | Dundalk | -12.445 | <0.001 |
|  | Cork | -14.588 | <0.001 |
|  | Arcachon | -8.134 | <0.001 |
| Sporocysts | Arcachon | 2.048 | 0.040 |
| *Paravortex* | Dundalk | -3.451 | <0.001 |
|  | Cork | -3.109 | 0.002 |
|  | Arcachon | 5.183 | <0.001 |
| *G. minutus* | Cork | -5.226 | <0.001 |
|  | Arcachon | 2.865 | 0.004 |
| *Trichodina* | Cork | 2.377 | 0.017 |
|  | Arcachon | -3.368 | <0.001 |
| *Rhynchodida* | Arcachon | -2.194 | <0.001 |
