# Supplementary material for: Extrinsic and intrinsic drivers of parasite prevalence and parasite species richness in a marine bivalve
Source: PLoS One. 2022 Sep 26;17(9):e0274474. doi: 10.1371/journal.pone.0274474 (PMC9512183; doi:10.1371/journal.pone.0274474)
Supplement: S1 Fig — Slides were prepared using histological techniques and stained with Haematoxylin and Eosin. (A) Rickettsiae-like infection in the gill (B) Gregarines within a granuloma (C) Trichodina ciliate external to the gill (D) Trematode metacercariae in the foot (E) Gymnophallus minutus in the hinge tissue (F) Disseminated neoplasia in the connective tissue. (DOCX) [file pone.0274474.s007.docx]

**Supplementary Material: Extrinsic and intrinsic drivers of parasite prevalence and parasite species richness in a marine bivalve**

| 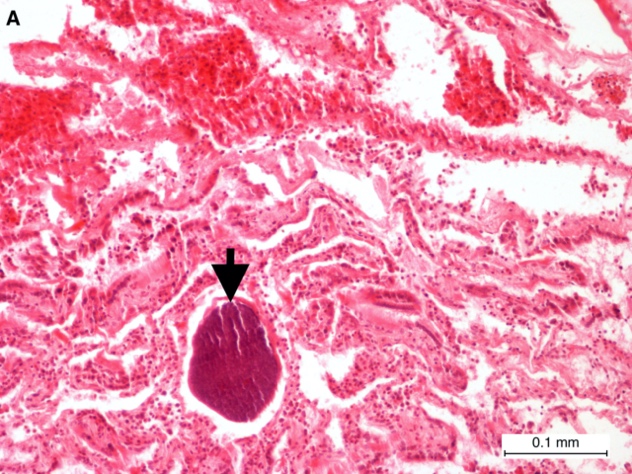 | 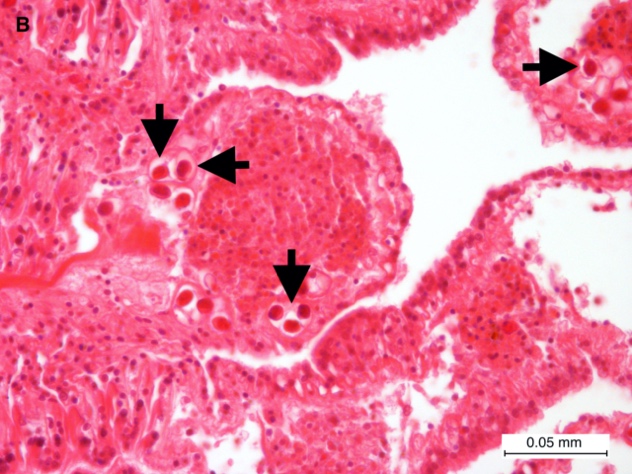 |
| --- | --- |
| 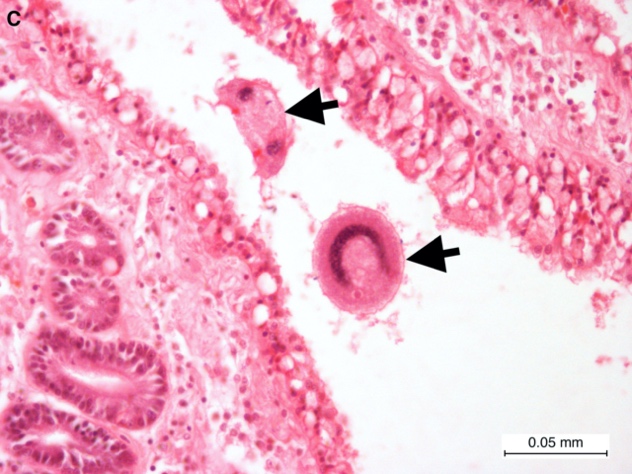 | 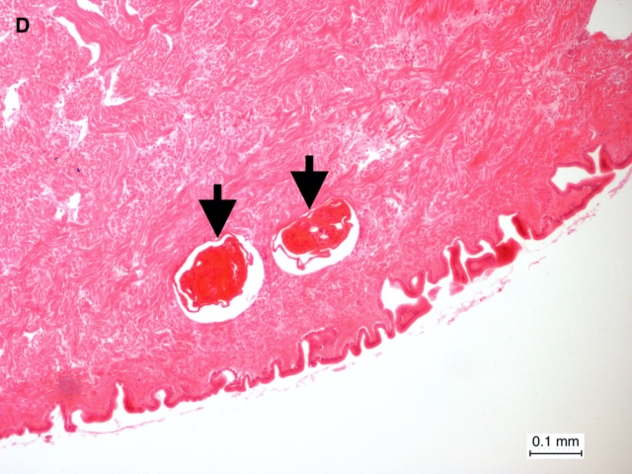 |
| 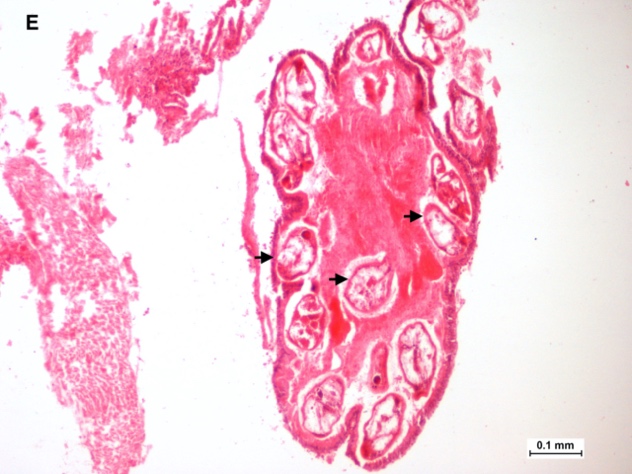 | 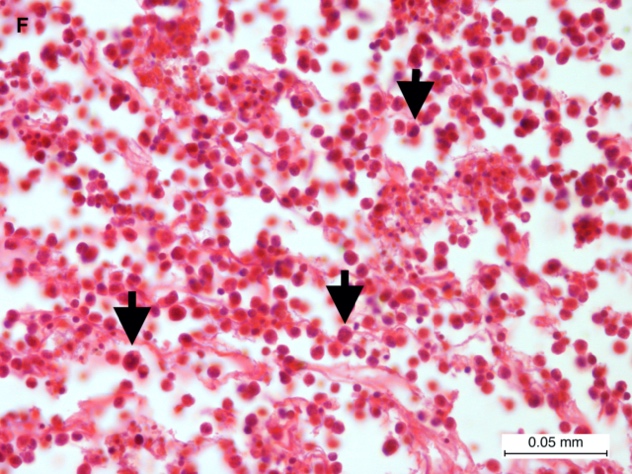 |

**S1 Figure**. **Examples of pathogens and pathologies (arrows) observed in *Cerastoderma edule* from Ireland and France, between April 2018 and October 2019.** Slides were prepared using histological techniques and stained with Haematoxylin and Eosin. (A) *Rickettsiae*-like infection in the gill (B) Gregarines within a granuloma (C) *Trichodina* ciliate external to the gill (D) Trematode metacercariae in the foot (E) *Gymnophallus minutus* in the hinge tissue (F) Disseminated neoplasia in the connective tissue.
