# Supplementary material for: Extrinsic and intrinsic drivers of parasite prevalence and parasite species richness in a marine bivalve
Source: PLoS One. 2022 Sep 26;17(9):e0274474. doi: 10.1371/journal.pone.0274474 (PMC9512183; doi:10.1371/journal.pone.0274474)
Supplement: S2 Fig — Mean ± 1 SD of temperature is represented by the vertical line and point within the violin plots. (DOCX) [file pone.0274474.s008.docx]

**Supplementary Material: Extrinsic and intrinsic drivers of parasite prevalence and parasite species richness in a marine bivalve**

**S2 Figure. Violin plots (visualising the kernel probability density), demonstrating the relationship between the presence of pathogens and seawater temperature, at each of the sites.** Mean ± 1 SD of temperature is represented by the vertical line and point within the violin plots.
